# Contribution to the knowledge of Galumnoidea (Acari, Oribatida) of Cuba

**DOI:** 10.3897/zookeys.537.6644

**Published:** 2015-11-18

**Authors:** Sergey G. Ermilov, Andrei V. Tolstikov

**Affiliations:** 1 Tyumen State University, Tyumen, Russia

**Keywords:** Oribatid mites, Galumnoidea, new species, systematics, morphology, supplementary description, new record, Cuba

## Abstract

An annotated checklist of identified oribatid mites of the superfamily Galumnoidea collected from Cuba, including ten species from four genera and two families, is provided. *Galumna flabellifera* Hammer, 1958, *Pergalumna bifissurata* Hammer, 1972, *Pergalumna bryani* (Jacot, 1934), *Pergalumna decorata* Balogh & Mahunka, 1977 and *Galumnopsis secunda* Sellnick, 1923 are recorded for the first time in the Cuban fauna. A new species of *Pergalumna*, *Pergalumna cubaensis* **sp. n.**, is described; it is morphologically similar to *Pergalumna decorata* Balogh & Mahunka, 1977, but differs from the latter by the larger body size, heavily granulated prodorsum and well-developed interlamellar setae. The adult of *Allogalumna cubana* Balogh & Mahunka, 1979 is redescribed.

## Introduction

At present, oribatid mites of the superfamily of Galumnoidea (Acari, Oribatida) are poorly known in the Cuban fauna (Balogh and Mahunka 1979; Jeleva et al. 1984; Palacios-Vargas and Socarrás 1993; Socarrás and Palacios-Vargas 1999). During taxonomic identification of material collected from Cuba, ten galumnoid species were found, including one new for science. The main goal of the paper is to describe this species under the name *Pergalumna cubaensis* sp. n.

The genus *Pergalumna* is a large genus with more than 140 species having a cosmopolitan distribution (Subías 2004, updated 2015). The updated generic diagnosis and identification key to known species in the Neotropical region were presented by Ermilov et al. (2013, 2014b).

Additionally, data are presented on the specific localities, with notes on new records, overall known distributions of registered taxa, and a supplementary description of *Allogalumna cubana* Balogh & Mahunka, 1979, which was described briefly and incompletely by Balogh and Mahunka (1979) from Cuba.

## Material and methods

These results are based on collections from three localities in Cuba (unknown date and collector, mites were previously deposited in the Museum of Zoology of Tyumen State University, Russia):

- Cuba 1: Parque Nacional Alejandro de Humboldt, 20°30'N, 74°40'W, leaf litter in forest.
- Cuba 2. Cuba, Valle de Viñales National Park, 22°40'56.8"N, 83°42'57.5"W, Ancon, leaf litter in forest.
- Cuba 3: Cayo Santa Maria, 22°66'21"N, 78°96'88"W, leaf litter in forest.

Specimens were mounted in lactic acid on temporary cavity slides for measurement and illustration. The body length was measured in lateral view, from the tip of the rostrum to the posterior edge of the ventral plate. Notogastral width refers to the maximum width in dorsal aspect. Lengths of body setae were measured in lateral aspect. All body measurements are presented in micrometers. Formulas for leg setation are given in parentheses according to the sequence trochanter–femur–genu–tibia–tarsus (famulus included). Formulas for leg solenidia are given in square brackets according to the sequence genu–tibia–tarsus. General terminology used in this paper follows that of Grandjean (summarized by Norton and Behan-Pelletier 2009). Drawings were made with a camera lucida using a Carl Zeiss transmission light microscope “Axioskop-2 Plus”.

## Systematics

### Pergalumna cubaensis sp. n.

Taxon classificationAnimaliaSarcoptiformesGalumnidae

http://zoobank.org/76C9BD7F-380A-43C5-8783-B4ADB318CF20

Figs 1–2 , 3–4 , 5–11

#### Diagnosis.

Body size: 962–1029 × 763–780. Prodorsum, epimeral region and antero-lateral parts of pteromorphs heavily granulated. Notogaster, anogenital region, pteromorphs and genital and anal plates striate. Rostral, lamellar, interlamellar and bothridial setae setiform, slightly barbed. Anterior notogastral margin well-developed. Three pairs of porose areas (*Aa*, *A2*, *A3*) rounded. Median pore and postanal porose area absent.

#### Description.

*Measurements*. Body length: 1012 (holotype: female), 962, 1029 (two paratypes: female and male); notogaster width: 763 (holotype), 763, 780 (two paratypes).

*Integument*. Body color black-brownish. Prodorsum, epimeral region and antero-lateral parts of pteromorphs heavily granulated; granules rounded or slightly elongated, their diameter or length up to 6. Notogaster, anogenital region, pteromorphs and genital and anal plates striate.

*Prodorsum*. Rostrum broadly rounded. Lamellar (*L*) and sublamellar (*S*) lines distinct, parallel, curving backwards. Rostral (*ro*, 77–86) and lamellar (*le*, 53–65) setae thin, slightly barbed, directed antero-medially. Interlamellar setae (*in*, 86–90) setae setiform, indistinctly barbed, directed medially. Bothridial setae (*bs*, 110–123) setiform, slightly barbed, directed postero-laterad. Exobothridial setae and their alveoli absent. Porose areas *Ad* absent.

*Notogaster*. Anterior notogastral margin well developed. Dorsophragmata (*D*) of medium size, elongated longitudinally. Notogastral setae represented by ten pairs of alveoli. Three pairs of porose areas (*Aa*, *A2*, *A3*) rounded, similar in diameter (20–24), with clear borders. Areas *Aa* located between setal alveoli *la* and *lm*, equal distanced from them. Median pore absent in male and females. All lyrifissures (*ia*, *im*, *ip*, *ih*, *ips*) distinct, *im* and opisthonotal gland openings (*gla*) located antero-laterally to *A2*.

*Gnathosoma*. Morphology of subcapitulum, palps and chelicerae typical for *Pergalumna* (Engelbrecht 1972; Ermilov and Anichkin 2011; Ermilov et al. 2014a). Subcapitulum size: 200–205 × 196–200. Subcapitular setae setiform, slightly barbed, *a* (36–41) longer than *m* (28–32) and *h* (24–28); *a* thickest, *h* thinnest. Two pairs of adoral setae (*or*_1_, *or*_2_, 24–28) setiform, barbed. Palp length: 176. Axillary sacculi (*sac*) distinct. Chelicera length: 303. Cheliceral setae setiform, barbed, *cha* (106) longer than *chb* (61).

*Epimeral and lateral podosomal regions*. Anterior tectum of epimere I smooth. Setal formula: 1–0–2–3. Setae thin, slightly barbed, *1b*, *3b*, *3c* and *4c* (41–49) longer than *4a* and *4b* (24–28) Pedotecta II trapezoid in ventral view. Discidia sharply triangular. Circumpedal carinae (*cp*) reaching insertions of *3b*.

*Anogenital region*. Six pairs of genital (*g*_1_, *g*_2_, 36–45; *g*_3_–*g*_6_, 20–28), one pair of aggenital (*ag*, 20–28), two pairs of anal (*an*_1_, *an*_2_, 20–28) and three pairs of adanal (*ad*_1_–*ad*_3_, 20–28) setae thin, indistinctly barbed. Genital plates with two genital setae on anterior edge. Adanal lyrifissures (*iad*) located diagonally to anal plates. Distance *ad*_1_–*ad*_2_ shorter than *ad*_2_–*ad*_3_. Setae *ad*_3 _inserted laterally to *iad.* Postanal porose area absent.

*Legs*. Morphology of leg segments, setae and solenidia typical for *Pergalumna* (see Engelbrecht 1972; Ermilov and Anichkin 2011; Ermilov et al. 2014a). Tridactylous, claws smooth. Formulas of leg setation and solenidia: I (1–4–3–4–20) [1–2–2], II (1–4–3–4–15) [1–1–2], III (1–2–1–3–15) [1–1–0], IV (1–2–2–3–12) [0–1–0]; homology of setae and solenidia indicated in Table 1. Solenidion φ of tibiae IV inserted dorsally at about 2/3 length of segment.

**Figures 1–2.**
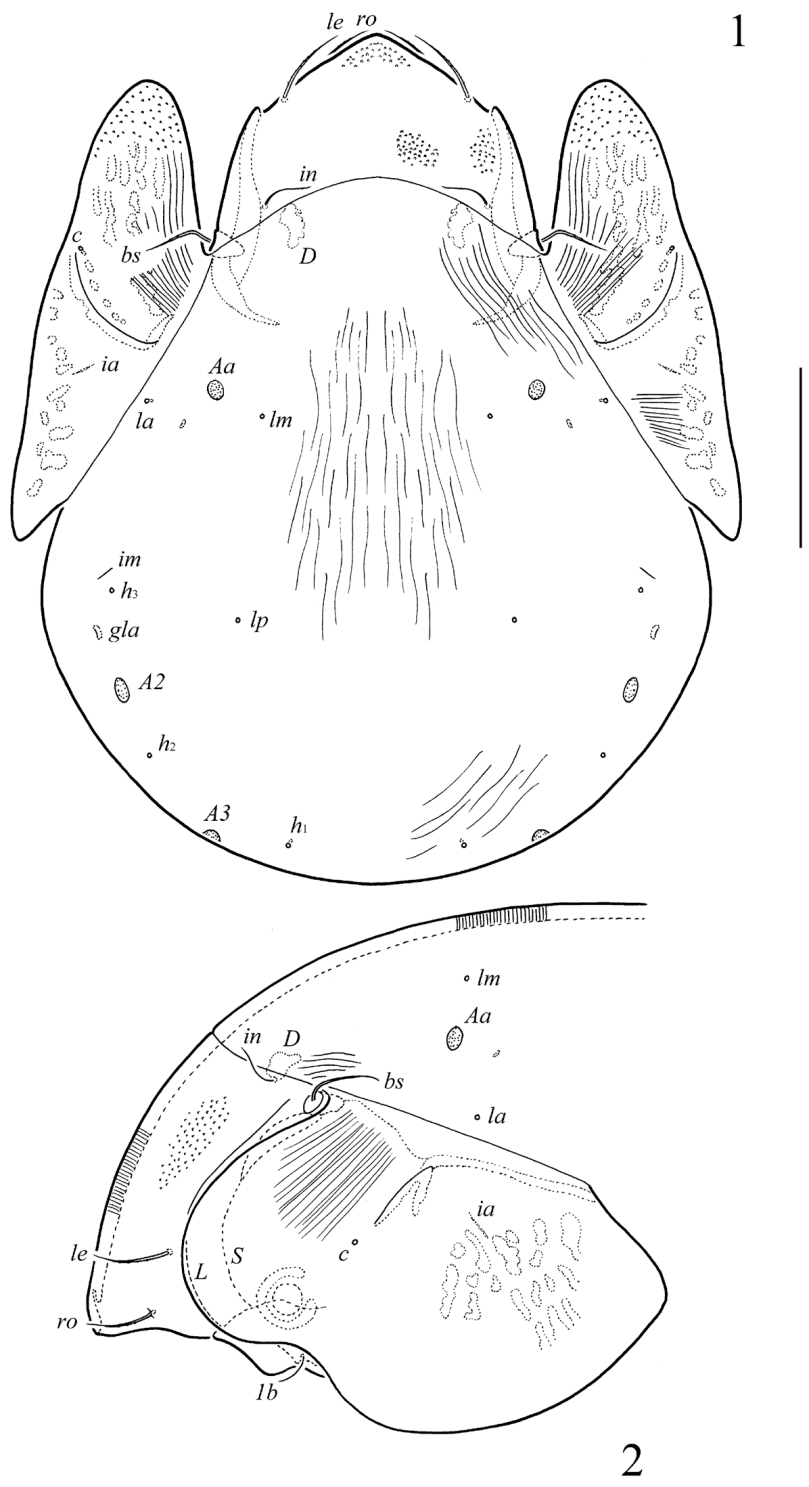
*Pergalumna cubaensis* sp. n., adult: **1** dorsal view (striae and granules are shown partially) **2** anterior part of body, lateral view (gnathosoma and leg I not illustrated, striae and granules are shown partially). Scale bar 200 µm.

**Figures 3–4.**
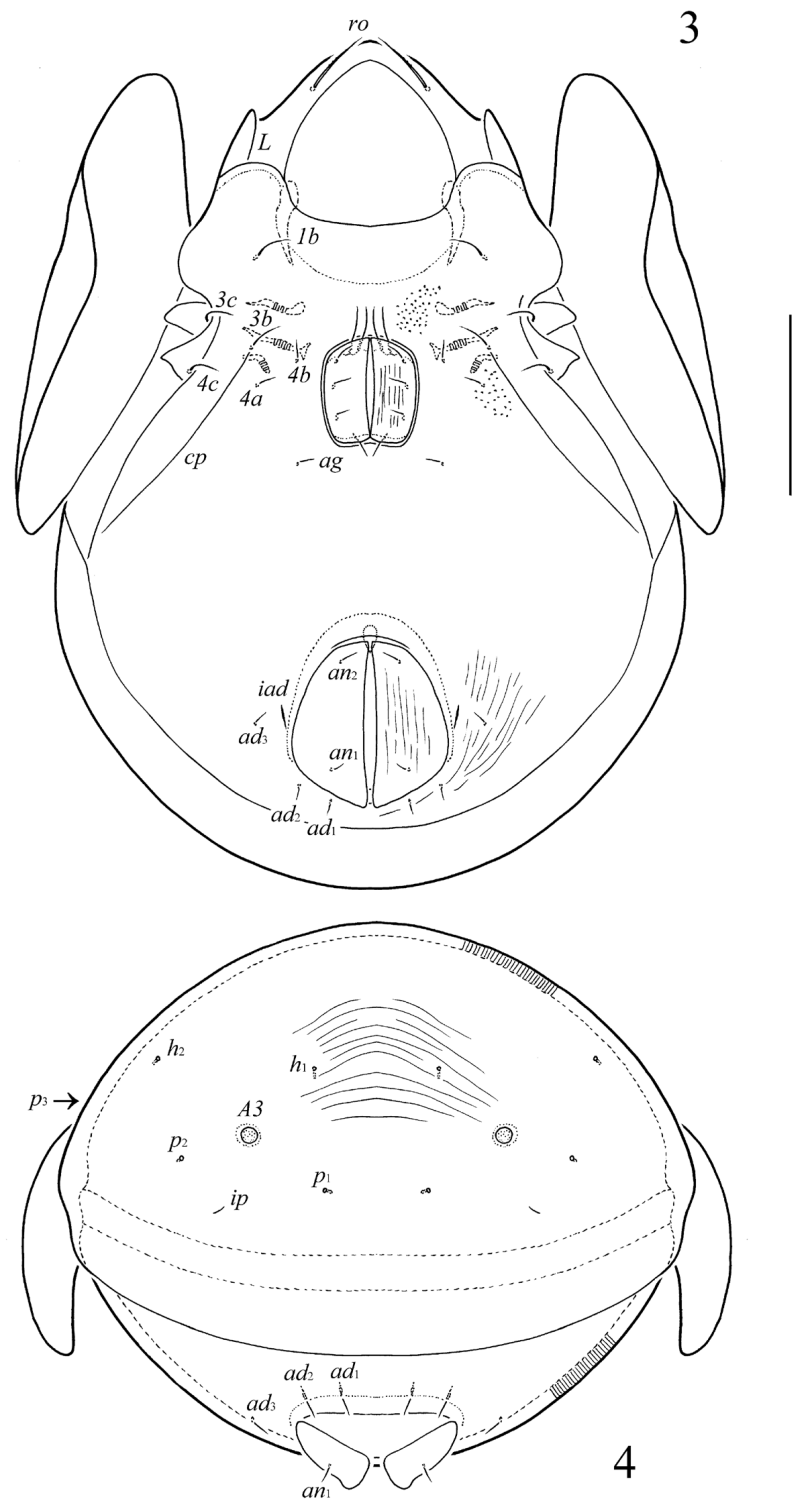
*Pergalumna cubaensis* sp. n., adult: **3** ventral view (gnathosoma and legs not illustrated, striae and granules are shown partially) **4** posterior view. Scale bar 200 µm.

**Figures 5–11.**
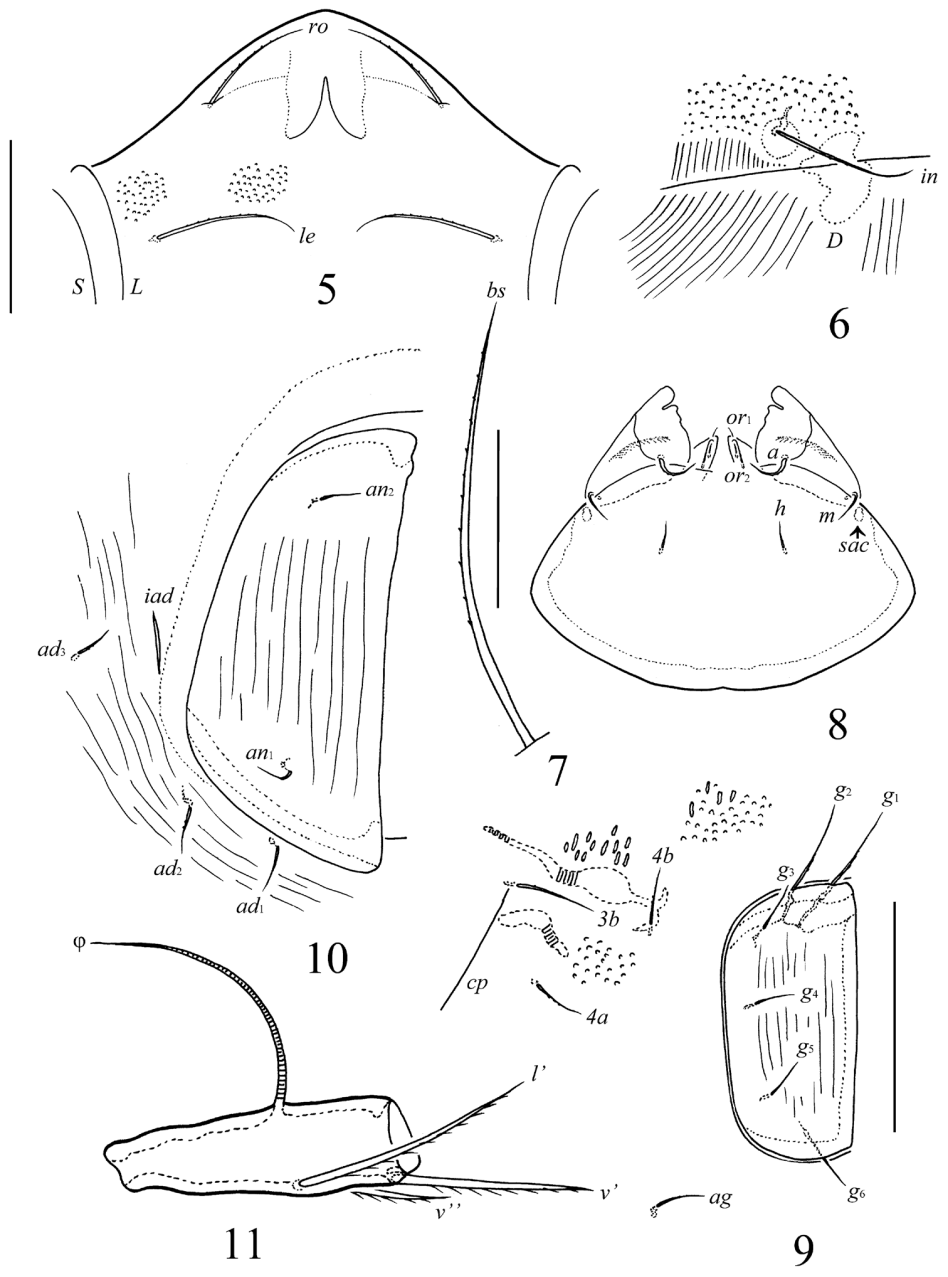
*Pergalumna cubaensis* sp. n., adult: **5** rostrum, frontal view (granules are shown partially) **6** interlamellar seta and part of sejugal region **7** bothridial seta **8** subcapitulum (in dissected specimen), ventral view **9** right genital plate and part of epimeral and aggenital regions (granules are shown partially) **10** right anal plate and part of adanal region **11** tibia of leg IV, left, antiaxial view. Scale bars 100 µm (**5, 6, 8–11**), 50 µm (**7**).

**Table 1.** Leg setation and solenidia of adult *Pergalumna cubaensis* sp. n. (same data for *Allogalumna cubana* Balogh & Mahunka, 1979).

Leg	Tr	Fe	Ge	Ti	Ta
I	*v*’	*d*, (*l*), *bv*’’	(*l*), *v*’, σ	(*l*), (*v*), φ_1_, φ_2_	(*ft*), (*tc*), (*it*), (*p*), (*u*), (*a*), *s*, (*pv*), *v*’, (*pl*), *l*’’, ε, ω_1_, ω_2_
II	*v*’	*d*, (*l*), *bv*’’	(*l*), *v*’, σ	(*l*), (*v*), φ	(*ft*), (*tc*), (*it*), (*p*), (*u*), (*a*), *s*, (*pv*), ω_1_, ω_2_
III	*v*’	*d*, *ev*’	*l*’, σ	*l*’, (*v*), φ	(*ft*), (*tc*), (*it*), (*p*), (*u*), (*a*), *s*, (*pv*)
IV	*v*’	*d*, *ev*’	*d*, *l*’	*l*’, (*v*), φ	*ft*’’, (*tc*), (*p*), (*u*), (*a*), *s*, (*pv*)

Note: Roman letters refer to normal setae, Greek letters to solenidia (except ε = famulus). Single prime (‘) marks setae on the anterior and double prime (“) setae on the posterior side of a given leg segment. Parentheses refer to a pair of setae. Tr – trochanter, Fe – femur, Ge – genu, Ti – Tibia, Ta – tarsus.

#### Material examined.

Locality Cuba 1: holotype (female) and two paratypes (female and male).

#### Type deposition.

The holotype is deposited in the collection of the Senckenberg Museum, Görlitz, Germany; two paratypes are in the collection of the Tyumen State University Museum of Zoology, Tyumen, Russia.

#### Etymology.

The specific name *cubaensis* refers to the country of origin, Cuba.

#### Remarks.

*Pergalumna cubaensis* sp. n. is morphologically most similar to *Pergalumna decorata* Balogh & Mahunka, 1977 from the Neotropical region (see Balogh and Mahunka 1977) in having a rounded rostrum, a striate notogaster, an anterior margin of notogaster, three pairs of rounded porose areas on the notogaster, and setiform bothridial setae. However, the new species differs from the latter by the larger body size (962–1029 × 763–780 *vs.* 637–653 × 469–494 in *Pergalumna decorata*), a heavily granulated prodorsum (*vs.* striate in *Pergalumna decorata*), and the interlamellar setae being of medium size (*vs.* minute in *Pergalumna decorata*).

### Allogalumna cubana

Taxon classificationAnimaliaSarcoptiformesGalumnidae

Balogh & Mahunka, 1979

Figs 12–13 , 14–15 , 16–23

#### Supplementary description.

*Measurements*. Body length: 332–348 (12 specimens: six females and six males); notogaster width: 232–249 (12 specimens). Without sexual dimorphism.

*Integument*. Body color brown to light brown. Body surface punctate (visible under high magnification, ×1000).

*Prodorsum*. Rostrum broadly rounded. Sublamellar lines distinct, curving backwards. Rostral (12–16) and lamellar (6–8) setae thin, smooth, directed antero-medially. Interlamellar setae minute (2). Bothridial setae (65–73) with elongated, unilaterally dilated and sparsely ciliated head, directed postero-laterad. Exobothridial setae and their alveoli absent. Porose areas *Ad* oval, transversally oriented (8 × 4), usually visible only in dissected specimens.

*Notogaster*. Anterior notogastral margin not developed. Dorsophragmata of medium size, elongated longitudinally. Notogastral setae represented by 10 pairs of alveoli. Four pairs of porose areas without clear borders: *Aa* oval, slightly transversally oriented (32–41 × 20), but it seems round in dorsal view; *A1* rounded (12–16); *A2* (16–20 × 10–12) and *A3* (16–24 × 12–16) oval. Areas *Aa* located antero-medially to *la*. Often small additional porose parts (*Aad*; one to three represented by five to nine heavily pores) present nearly of *Aa*, but they visible only high magnification (Fig. 19). Median pore absent in males and females. All lyrifissures distinct, *im* located between *lm* and *A1*. Opisthonotal gland openings located laterally to *A1*.

*Gnathosoma*. Morphology of subcapitulum, palps and chelicerae similar to *Pergalumna cubaensis* sp. n. Subcapitulum size: 86–90 × 82–86. Subcapitular setae setiform, slightly barbed, *a* (14–16) longer than *m* (10–12) and *h* (8); *a* thickest, *h* thinnest. Two pairs of adoral setae (8) setiform, barbed. Palp length: 69. Axillary sacculi distinct. Chelicera length: 127. Cheliceral setae setiform, barbed, *cha* (32) longer than *chb* (20).

*Epimeral and lateral podosomal regions*. Anterior tectum of epimere I smooth. Setal formula: 1–0–1–2. Setae thin, smooth, *1a*, *3b* and *4a* (10) longer than *4b* (6) Pedotecta II rectangular, rounded distally in ventral view. Discidia sharply triangular. Circumpedal carinae clearly not reaching insertions of *3b*.

*Anogenital region*. Six pairs of genital (*g*_1_, *g*_2_, 10; *g*_3_–*g*_6_, 6), one pair of aggenital (4), two pairs of anal (4) and three pairs of adanal (4) setae thin, smooth. Genital plates with two genital setae on anterior edge. Adanal lyrifissures located parallel to anal plates. Distance *ad*_1_–*ad*_2_ shorter than *ad*_2_–*ad*_3_. Setae *ad*_3 _inserted laterally to *iad.* Postanal porose area oval, transversally oriented (12–16 × 6–10).

*Legs*. Morphology of leg segments, setae and solenidia, formulas of leg setation and solenidia similar to *Pergalumna cubaensis* sp. n. (Table 1), but solenidion φ of tibiae IV inserted dorsally at about 1/3 length of segment, directed backwards in basal part.

**Figures 12–13.**
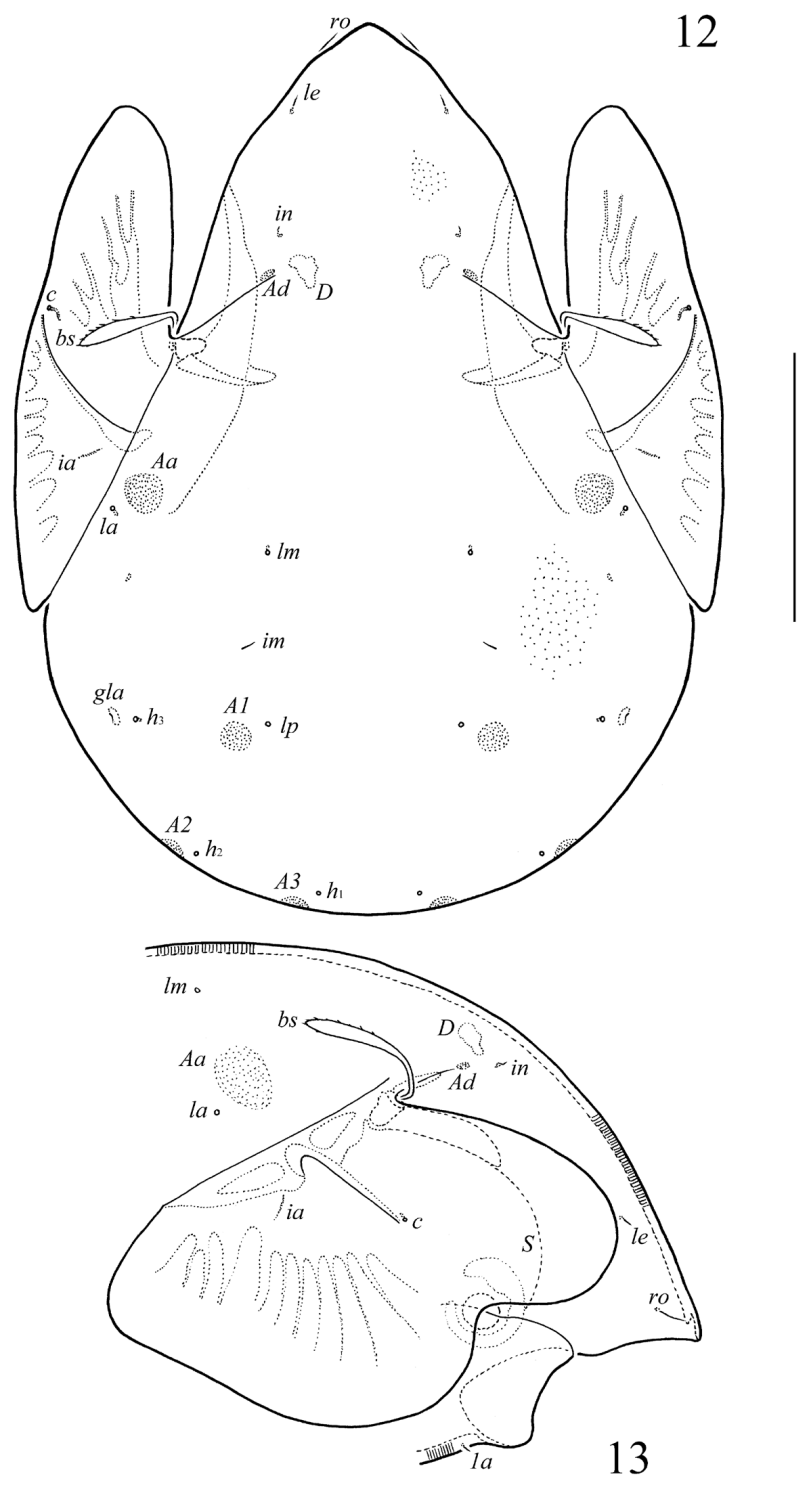
*Allogalumna cubana* Balogh & Mahunka, 1979, adult: **12** dorsal view (microfoveolae are shown partially) **13** anterior part of body, lateral view (gnathosoma and leg I not illustrated). Scale bar 100 µm.

**Figures 14–15.**
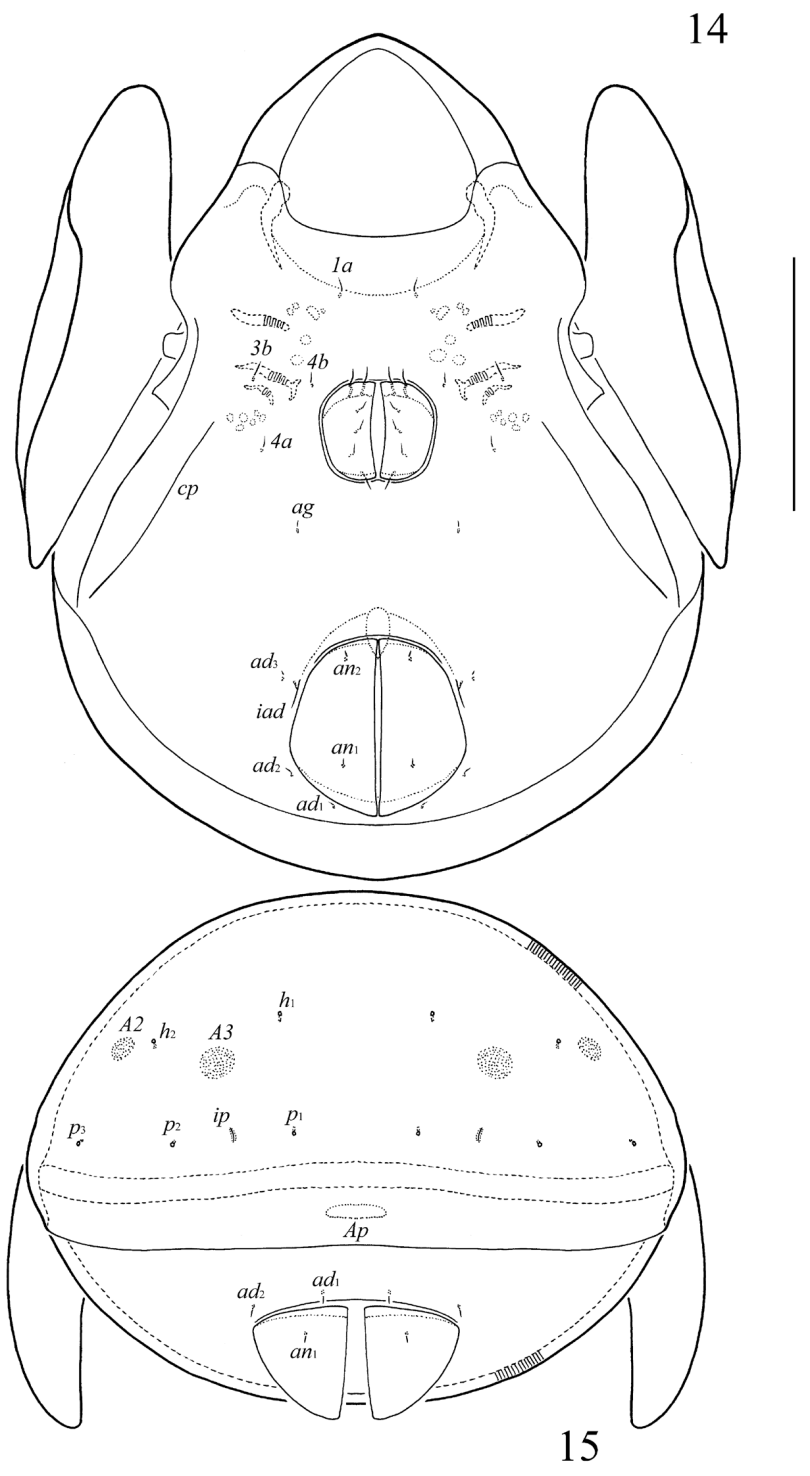
*Allogalumna cubana* Balogh & Mahunka, 1979, adult: **14** ventral view (gnathosoma and legs not illustrated) **15** posterior view. Scale bar 100 µm.

**Figures 16–23.**
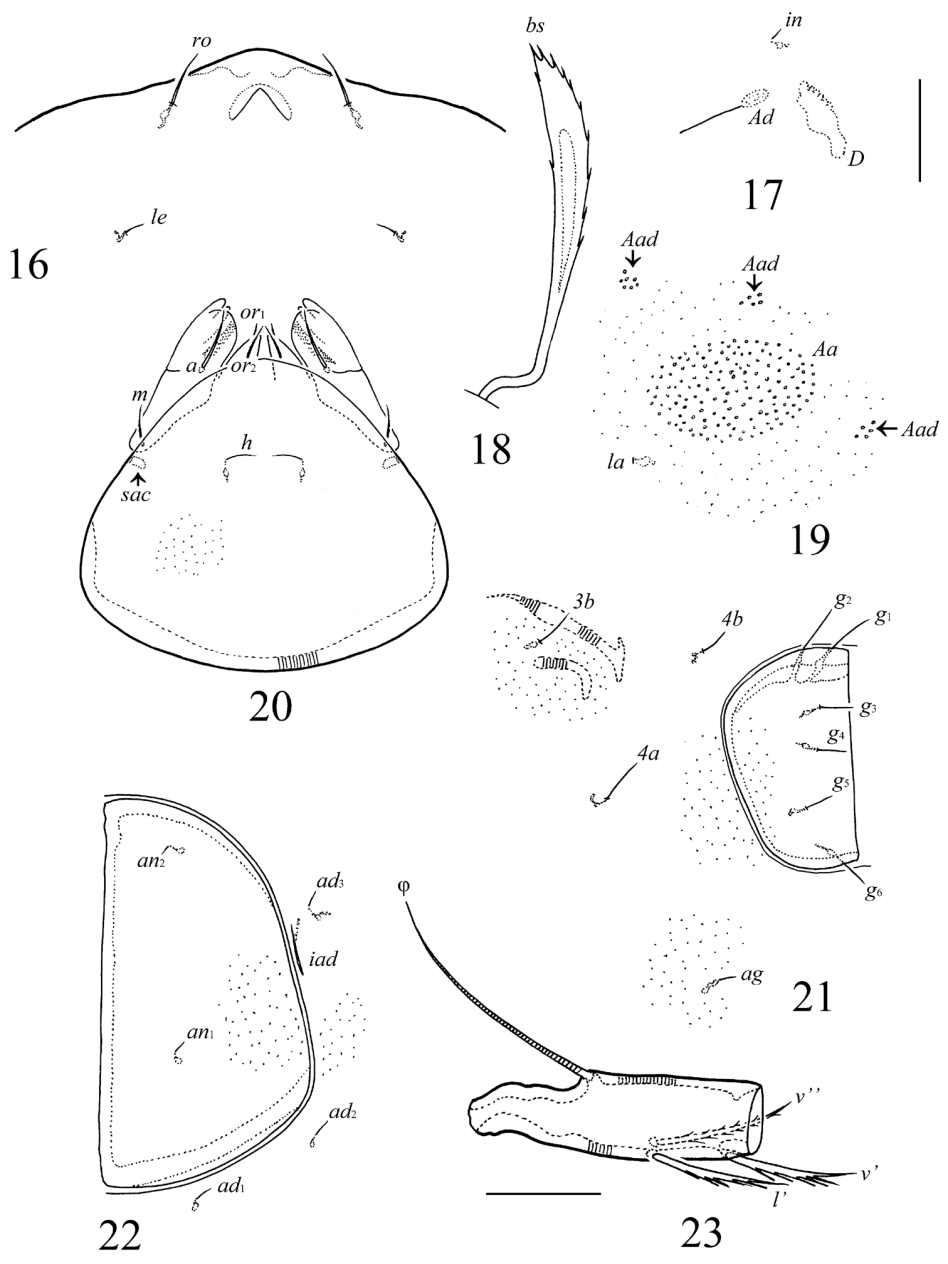
*Allogalumna cubana* Balogh & Mahunka, 1979, adult: **16** rostrum, frontal view **17** interlamellar seta and part of sejugal region **18** bothridial seta **19** left setal alveolus *c* and porose area *Aa* with additional areas **20** subcapitulum (in dissected specimen), ventral view (microfoveolae are shown partially) **21** right genital plate and part of epimeral and aggenital regions (microfoveolae are shown partially) **22** left anal plate and part of adanal region (microfoveolae are shown partially) **23** tibia of leg IV, left, antiaxial view. Scale bars 20 µm.

#### Material examined.

Locality Cuba 2: 12 specimens (six females and six males).

#### Remarks.

The Cuban specimens of *Allogalumna cubana* from Balogh and Mahunka’s description (1979) and our specimens are identical morphologically. Hence, based on these data, the main characters of *Allogalumna cubana* are: small body size (328–348 × 232–251); body surface indistinctly punctate; rostrum rounded; rostral setae longer than lamellar setae, all thin, smooth; interlamellar setae minute; bothridial setae with elongated, unilaterally dilated and sparsely ciliated head; anterior notogastral margin not developed; four pairs of oval/rounded porose areas, *Aa* slightly transversally oriented; median pore absent; epimeral and anogenital setae thin, smooth; setae *ad*_3 _inserted laterally to *iad*; postanal porose area present; tridactylous.

### Records

*Galumna angularis* Jeleva, Scull & Cruz, 1984 (see Jeleva et al. 1984; Mahunka 1985; Pérez-Íñigo and Baggio 1994). Distribution: Neotropical region.

**Material examined.** Locality Cuba 1: 11 specimens.

*Galumna flabellifera* Hammer, 1958 (see Hammer 1958; Aoki 1964, 1982; Mahunka 1978). Distribution: Pantropical and Subtropical regions. New record in Cuba.

**Material examined.** Locality Cuba 2: 16 specimens.

*Galumna* sp. Species is morphologically similar to *Galumna lunaris* Jeleva, Scull & Cruz, 1984 (see Jeleva et al. 1984).

**Material examined.** Locality Cuba 3: 4 specimens.

**Remarks.** Jeleva et al. (1984) unclearly described *Galumna lunaris* from Cuba, therefore we could not identify our species without studying of the type material.

*Pergalumna bifissurata* Hammer, 1972 (see Hammer 1972; Ermilov et al. 2014). Distribution: Polynesia and Neotropical region. New record in Cuba.

**Material examined.** Locality Cuba 1: 22 specimens.

*Pergalumna bryani* (Jacot, 1934) (see Jacot 1934; Hammer 1973). Distribution: Pacific Islands and Neotropical region. New record in Cuba.

**Material examined.** Locality Cuba 2: 5 specimens.

*Pergalumna decorata* Balogh & Mahunka, 1977 (see Balogh and Mahunka 1977). Distribution: Neotropical region. New record in Cuba.

**Material examined.** Locality Cuba 1: 7 specimens.

*Pergalumna* sp. Species is morphologically similar to *Galumna brasiliensis* Sellnick, 1923 (see Sellnick 1923).

**Material examined.** Locality Cuba 1: 22 specimens; Locality Cuba 2: 18 specimens; Locality Cuba 3: 6 specimens.

**Remarks.** Sellnick (1923) briefly described several species of *Galumna* (including *Galumna brasiliensis*) from Brazil. To date, *Galumna brasiliensis* has not been redescribed in detail. Lamellar seta appear to be inserted medially to the lamellar line according to figure 27 in Sellnick (1923); therefore there is a probability, that *Galumna brasiliensis* is a representative of *Pergalumna*. Hence, the systematic position of *Galumna brasiliensis* should be investigated further.

**Galumnellidae**

*Galumnopsis secunda* Sellnick, 1923 (see Sellnick 1923). Distribution: Neotropical region. New record in Cuba.

**Material examined.** Locality Cuba 2: 5 specimens.

## Supplementary Material

XML Treatment for Pergalumna cubaensis

XML Treatment for Allogalumna cubana
